# Commentary: Turning to the arts to transform and humanise nursing research

**DOI:** 10.1177/17449871261465143

**Published:** 2026-08-12

**Authors:** Elizabeth Jestico

**Affiliations:** Senior Lecturer in Children’s Nursing, Oxford Brookes University, Oxford, UK

**Commentary to:** Poetry as reflexive lens: humanising nursing practice in robotic-assisted surgery (Lee, 2026)

It was an absolute joy to read Lee’s (2026) paper about how she used poetry in her doctoral study exploring nursing practice in robotic-assisted surgery. Lee’s use of poetry draws out the emotion of both the participants’ and the researcher’s experience, and so my initial emotional response of joy feels significant. I am sure that other readers will also experience an emotional response to this work. Poetry and other art forms hold the potential to push the frontiers of nursing research, allowing us as the audience to ‘experience’ research, rather than just gain knowledge and understanding. It is this experiential and emotional response that allows research to become humanised, and this is a place we can only truly reach by pushing frontiers and merging boundaries.

A few weeks ago I was in Paris, and whilst wandering around an art gallery, I became captivated by a Monet painting. What struck me was the fusion of boundaries between the object (a willow tree) and its reflection in water. I started to ponder this as a metaphor for research and indeed for life. We experience life with an awareness that there is a boundary between events and our interpretations of these, but this often remains virtually imperceptible. The process of being reflexive though, can help us navigate around and through that fuzzy boundary. As Lee (2026) beautifully articulates, poetry can equip us with a reflective lens where we can dwell in the intersection between participants’ stories and our interpretations of these.

Yet the role of poetry in research can take us far beyond the task of being reflexive. Darbyshire and Oerther (2021: 524) also turned to poetry in their research and argued that the arts can shape our thinking in a different way from standard academic prose and help us to access a way of ‘meditative thinking’. This allows us to engage at a different level and become more attuned to people’s unique stories and support needs. I crafted a poem in my doctoral study (Jestico et al., 2026) and noticed that as I have presented this at conferences and events, the audiences have experienced visible emotional responses. I realised that the poem was able to evoke resonance and a connection with participants’ experiences in a raw and emotive way. Poetry can humanise and breathe life into research, and this is why it enables us to actually experience the research rather than just intellectually engage with it. This is critical for nursing research, as when research feels alive, human and meaningful, it perhaps stands the greatest chance of being applied and translated to our nursing practice. The magic of this then is that having used poetry as a reflexive lens for the researcher it can, as proposed by Cleary et al. (2022), go on to become a catalyst for practitioners’ professional reflexivity.

One of the strengths of the arts is their limitlessness, and their application to nursing research can therefore open so many doors. In my doctoral study, I was able to draw on music and paintings as a sequence of metaphors for participants’ stories (Jestico, 2024). Like poetry, this acted as a powerful reflexive lens and similarly facilitated a connection with participants’ experiences beyond the usual academic discourse. But this leads me to consider where else the arts could take us in nursing research and practice. Much poetic and arts-based research operates within the sphere of qualitative methodologies, but I wonder what role they could play in quantitative approaches. Acknowledging that the arts can help us connect with and understand the experiences of people who we care for, I also wonder what role could (or should) the arts play in nursing education. And finally, I wonder how much more the arts could be used as an inherent part of what we do in practice, and who we are as nurses.

Lee (2026) wrote about the ‘art of nursing’. Nursing has long been regarded as both an art and science (Darbyshire, 1999), so as Lee argued, perhaps we should feel more comfortable with the concept of nursing research also being an art as well as a science. If we can be open to this and allow science and art to collide, and if we can find a space where the arts do not replace scientific rigour but complement and enhance it, then we create a world where in both academia and practice, we can emotionally engage with true reflexivity and humanity. I found that engaging with the arts in my research led to a deep and profound transformation within me, both professionally and personally. Whilst it is not perhaps possible to capture impact like this in a measurable way, the importance of transformation like this cannot be underestimated. This is where the magic and life of research exists which, in Lee’s (2026: 3) poetic words can take us all on ‘a transformative journey’.

## References

[bibr1-17449871261465143] Cleary B Carnevale F Tsimicalis A (2022) Poetics of brittle bone disease: Using found poetry to explore childhood bioethics. Journal of Poetry Therapy 35: 241–258. DOI: 10.1080/08893675.2022.2043120.

[bibr2-17449871261465143] Darbyshire P (1999) Nursing, art and science: Revisiting the two cultures. International Journal of Nursing Practice 5: 123–131. DOI: 10.1046/j.1440-172x.1999.00127.x. 10769620

[bibr3-17449871261465143] Darbyshire P Oerther S (2021) Heidegger and parenthood: A theoretical and methodological shift from instrumental to ontological understanding. Journal of Child Health Care 25: 523-533. DOI: 10.1177/1367493520965836. 33021840

[bibr4-17449871261465143] Jestico E (2024) Shaping confidence to advocate for my child: The meaning of how ‘significant others’ can support parents to make decisions when their child has cancer. PhD Thesis, Oxford School of Nursing and Midwifery, Faculty of Health and Life Sciences, Oxford Brookes University. DOI: 10.24384/xhj7-y804.

[bibr5-17449871261465143] Jestico E Finlay T Schutz S (2026) ‘We are lost at sea’: How ‘significant others’ may shape parents’ confidence to make decisions when their child has cancer. Qualitative Health Research. Epub ahead of print. DOI: 10.1177/10497323261439567. 42165318

[bibr6-17449871261465143] Lee L (2026) Poetry as reflexive lens: humanising nursing practice in robotic-assisted surgery. Journal of Research in Nursing. Epub ahead of print 20 January 2026. DOI: 10.1177/17449871251407831. PMC12867712 41648834

